# *Stigmatopora harastii*, a new species of pipefish in facultative associations with finger sponges and red algae from New South Wales, Australia (Teleostei, Syngnathidae)

**DOI:** 10.3897/zookeys.994.57160

**Published:** 2020-11-17

**Authors:** Graham Short, Andrew Trevor-Jones

**Affiliations:** 1 Australian Museum Research Institute, Australian Museum, 1 William Street, Sydney NSW 2010, Australia Australian Museum Sydney Australia; 2 California Academy of Sciences, 55 Music Concourse Drive, San Francisco, CA 94118, USA California Academy of Sciences San Francisco United States of America; 3 Burke Museum of Natural History and Culture, 4300 15th Ave NE, Seattle, WA 98105, USA Burke Museum of Natural History and Culture Seattle United States of America; 4 IUCN Seahorse, Pipefish & Seadragon Specialist Group (SPS SG), Institute for the Oceans and Fisheries, The University of British Columbia 2202 Main Mall, Vancouver BC V6T 1Z4, Canada University of British Columbia Vancouver Canada

**Keywords:** Botany Bay, COI, cryptobenthic, ichthyology, Jervis Bay, marine fish, morphology, South Pacific, Sydney, systematics, taxonomy

## Abstract

A new species of pipefish, *Stigmatopora harastii***sp. nov.**, is described based on the male holotype and two female paratypes, 136.3–145.5 mm SL, collected from red algae (sp.?) at 12 meters depth in Botany Bay, New South Wales (NSW), Australia. The new taxon shares morphological synapomorphies with the previously described members of *Stigmatopora*, including principle body ridges, fin placement, slender tail, and absence of a caudal fin. It is morphologically and meristically similar to *Stigmatopora nigra*, including snout length and shape, dorsal-fin origin on 6^th^–7^th^ trunk ring, and lateral trunk ridge terminating on the first tail ring. *Stigmatopora harastii***sp. nov.** is distinguished from its congeners, however, by characters of the head and first trunk ring, distinct sexual dimorphic markings on sides and venter of anterior trunk rings, and red background coloration in life. The new taxon can be further differentiated by genetic divergence in the mitochondrial COI gene (uncorrected p-distances of 9.8%, 10.1%, 10.7%, and 14.6%, from *S. argus*, *S. macropterygia*, *S. narinosa*, and *S. nigra*, respectively). The type locality is characterised by semi-exposed deep-water sandy areas interspersed with boulders, flat reefs, and an absence of seagrass beds, in which *S. harastii* has been observed living in facultative associations with a finger sponge and red algae at depths of 10–25 meters, compared to the shallow coastal and estuarine habitats preferred by the fucoid algae and seagrass-associating members of *Stigmatopora*. *Stigmatopora harastii***sp. nov.** represents the fourth species of *Stigmatopora* recorded in temperate southern Australia.

## Introduction

The Syngnathidae contains over 300 species within 57 genera of predominantly small-bodied and cryptic marine fishes (Dawson 1985; Fricke et al. 2020; WoRMS Editorial Board 2020). The pipefishes assigned to *Stigmatopora* Kaup, 1853 currently comprise four species, which are restricted to southern Australia and New Zealand: *S. argus* Richardson, 1840, from New South Wales (NSW) to Western Australia, including Tasmania, and New Zealand; *S. nigra* Kaup, 1856, from Mooloolaba, Queensland to Shark Bay, Western Australia, including around Tasmania, and New Zealand; *S. macropterygia* Duméril, 1870 from New Zealand; and *S. narinosa* Browne & Smith, 2007, a South Australian endemic. The genus is distinguished from other family members by a combination of features that include fin placement, slender distally coiled prehensile tail, and absence of caudal fin. Dawson (1982) provided valuable data for differentiating the species on the basis of meristic and morphometric characters and sexual dimorphism in the ventral trunk markings, which was further discussed by Browne and Smith (2007). Members of the *Stigmatopora* form an abundant component of the ichthyofauna of shallow vegetated coastal and estuarine habitats in southern Australia and New Zealand, in which they associate with various species of fucoid algae and seagrass (Dawson 1982; Pollard 1984; Howard and Koehn 1985; Bell et al. 1992; Steffe et al. 1992; Ferrell et al. 1993; Connolly 1994; Pollard 1994; Jenkins et al. 1997; Kendrick and Hyndes 2003; Browne and Smith 2007; Browne et al. 2008; Parkinson and Booth 2016).

The present paper describes a new species of *Stigmatopora* from NSW, Australia that was first reported by underwater photographers (Oceantrek Diving Resort) in Jervis Bay in 2002, and subsequently observed in Shellharbour and Botany Bay. *Stigmatopora harastii* occurrs in semi-exposed habitats consistent for the Sydney Basin bioregion (Andrew 1999), which is markedly different from the shallow coastal and estuarine habitats typically preferred by the fucoid algae and seagrass associating members of *Stigmatopora* (Dawson 1985; Steffe et al. 1989; Browne and Smith 2007; Parkinson and Booth 2016).

## Materials and methods

Type specimens (AMS I. 49510-001, holotype, male; AMS I.47267, paratypes, two females) are deposited in the collections of the Australian Museum (AM).

Counts and measurements to the nearest 0.1 mm were taken from high resolution digital images of specimens using ImageJ (Rasband et al. 1997). Head and body measurements and morphometrics follow Short et al. (2018). External morphological characters were documented using a dissecting microscope and analyses of high-resolution digital images. Georeferenced locations for the type specimens of *S. harastii* use dWGS84 datum and were captured on GPS units.

DNA extraction, primers and PCR conditions, sequence alignment, and analysis of COI sequence data were performed following protocols described in Hamilton et al. (2017). A partial segment of mitochondrial cytochrome c oxidase subunit I (COI) DNA was sequenced from a 95% ethanol-fixed eye sample from a paratype collected from the type locality (AMS I.47267-001). COI sequence data was compared to the previously sequenced *Stigmatopora* species *S. argus*, *S. macropterygia*, *S. narinosa*, and *S. nigra*, respectively (available from Hamilton et al. 2017) in order to calculate genetic distances (uncorrected P distances) in MEGA v. 7.0.26 (Kumar et al. 2017).

## Systematics

### Stigmatopora harastii sp. nov.

Taxon classificationAnimaliaSyngnathiformesSyngnathidae

2E993FAB-30F7-57FA-A16A-AB85494AD234

http://zoobank.org/CE61AAB9-3723-4B44-A025-1E8AE3486BD9

Figs 1 , 2 , 3 , 4 , 5 , 6 , 7 , 8 , 9 , 10

#### Type material.

***Holotype***: AMS I.49510-001, male, 145.5 mm SL, collected from a scuba dive area locally referred to as “The Steps”, Kamay Botany Bay National Park, Kurnell, Botany Bay, NSW, Australia, 34°00'07.9"S, 151°13'41.4"E, 13.5 m depth, 18 June 2020, by A. Trevor-Jones and D. Harasti.

***Paratypes***: AMS I.47267, two females, 130.7 mm and 135.2 mm SL, collected from a scuba dive area known as “The Steps”, Kamay Botany Bay National Park, Kurnell, Botany Bay, NSW, Australia, 34°00'07.9"S, 151°13'41.4"E, 12 m depth, 9 June 2017, by D. Harasti, R. Rodrigues, and A. Trevor-Jones.

#### Comparative material.

*Stigmatopora nigra* AMS I.42611-009, Botany Bay, NSW, Australia, 03 Feb 2003, K. Parkinson; *Stigmatopra narinosa*, SAMA F10190, holotype, 150 mm SL, South Australia, Edithburg Pool, 35°05'S, 137°45'E, 31 Dec 2003. Published data was obtained for *S. argus*, *S. macropterygia*, *S. narinosa*, and *S. nigra* from Dawson (1982, 1985).

#### Diagnosis.

*Stigmatopora harastii* differs from its congeners by the following combination of morphological characters: median ridge, distinct, low, present on dorsum of head and first trunk ring starting from the posterior third of the frontal, over the supraoccipital, to the anterior and posterior nuchal plates; opercular ridge prominent, complete, not angled dorsad; lateromedial ridge, distinct, low, present between opercle and pectoral fin base; dorsal-fin origin on 6^th^–7^th^ trunk rings, subdorsal rings 19–20 (12 trunk rings + 7 or 8 tail rings); lateral trunk ridge ends on first tail ring. Colouration: red background colour; dorsum of snout with large, irregular pale white spots; sides of head and anterior trunk rings with large, irregular pale white spots or with diffuse pale white stripe; venter of first trunk ring with distinct red elongated spots in longitudinal row, almost forming a stripe, on midline present in male (AMS I. 49510-001); venter of anterior trunk rings pale red with a large cluster of distinct red spots extending posteriad from second trunk ring in male (AMS I. 49510-001), few scattered small red spots in females (AMS I.1.47267).

#### Description.

**Figure 1.**
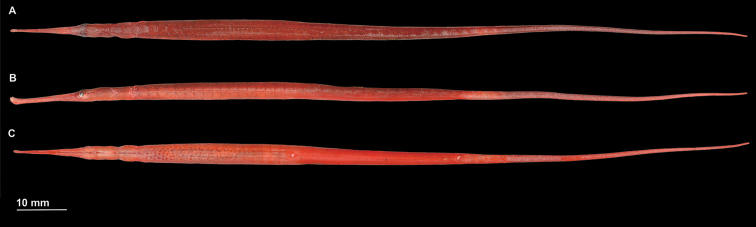
*Stigmatopora harastii*, preserved directly after collection, AMS I. 49510-001, holotype male, 145.5 mm SL**A** dorsal view **B** lateral view **C** ventral view; Australia: NSW, Botany Bay, Kurnell (photograph: Kerryn Parkinson).

**Figure 2.**
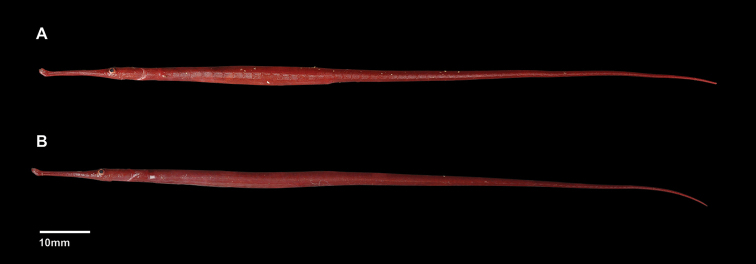
*Stigmatopora harastii*, preserved directly after collection, paratypes, female **A** AMS I.47267-001, 136.3 mm SL**B** AMS I.47267-002, 138.2 mm SL; Australia: NSW, Botany Bay, Kurnell (photograph: Kerryn Parkinson).

**Figure 3.**
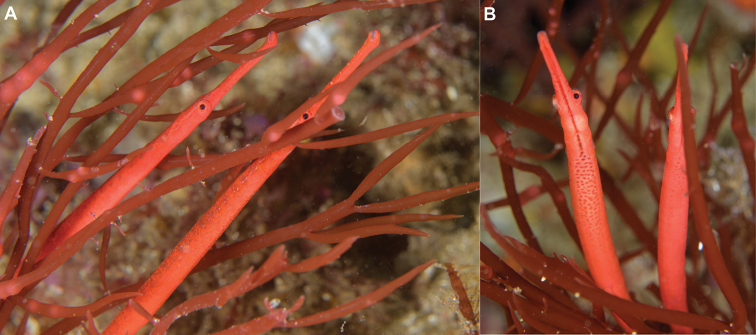
*Stigmatopora harastii* in situ, AMS I. 49510-001, holotype, male **A** (right individual) **B** (left individual); The Steps, Kurnell, Botany Bay, NSW, Australia, 13.5 meters depth, 18 June 2020. The male holotype was photographed with a paired female individual, which was not collected. Note the large cluster of distinct red spots extending posteriad on venter of anterior trunk rings in the male (photographs: Andrew Trevor-Jones).

**Figure 4.**
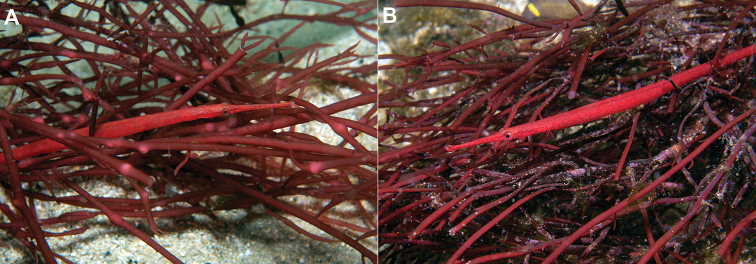
*Stigmatopora harastii* in situ, AMS I.47267 paratypes, female, The Steps, Kurnell, Botany Bay, NSW, Australia at 11–12 meters depth, 06 June 2017 (photographs: David Harasti).

**Figure 5.**
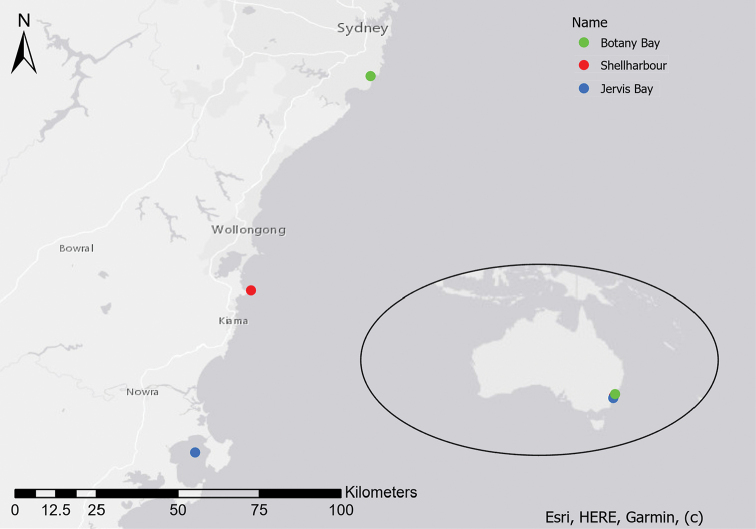
Distribution of *Stigmatopora harastii* in NSW, Australia. Type locality in green.

**Figure 6.**
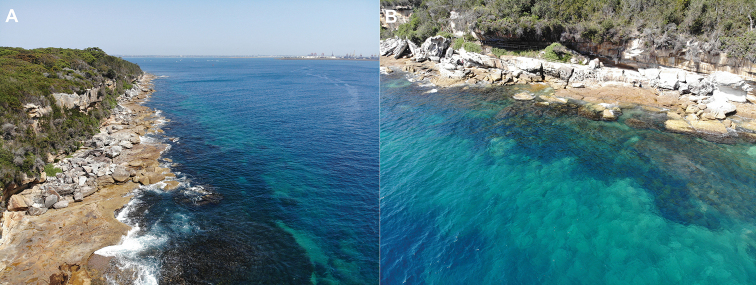
Aerial view of the scuba dive site The Steps, Kurnell, Botany Bay, NSW, Australia **A** shore and entrance **B** inshore boulders (photographs: Michael McFadyen).

General body shape as in Figs 1–6. Morphometric, meristic, and morphological characters listed in Table 1. Superior trunk and tail ridges continuous, not arched dorsad below the dorsal-fin base; lateral trunk ridge ends without deflection on the first tail ring; lateral tail ridge absent; inferior trunk and tail ridges continuous, the former largely located on the ventral portion of the trunk; dorsum of the trunk flat to slightly convex between superior ridges; trunk flat to slightly V-shaped ventrad, without a prominent median ridge; trunk compressed dorsoventrally and expanded laterad, especially in the females; tail slender, distally attenuated or thread-like. Snout long and slender; median dorsal snout ridge low, entire, failing to reach the interorbital, and ends just before vertical through nares; preorbital moderately broad, the nares well removed from anterior rim of orbit; interorbital broad, flat to slightly concave; median ridge, distinct, low, present on dorsum of head and first trunk ring starting from the posterior third of the frontal, over the supraoccipital, to the anterior and posterior nuchal plates; opercular ridge, prominent, complete, not angled dorsad; supraopercular ridges absent; opercle with or within a complete or incomplete longitudinal ridge, elsewhere ornamented with fine radiating striae; gill opening located above middle or posterior third of opercle; lateromedial ridge, distinct, low, present between cleithrum and pectoral fin base; principal body ridges low; head and body without spines, denticulations or dermal flaps; dorsal-fin origin on 6^th^–7^th^ trunk rings, subdorsal rings 19–20 (12 trunk rings + 7 or 8 tail rings); lateral trunk ridge ends on first tail ring; anal-fin rays four; pouch plates absent; brood pouch under anterior portion of tail; pouch plates absent.

**Table 1.** Selected counts and morphometric measurements for *Stigmatopora harastii*. Abbreviations: SnD (snout depth), SnL (snout length), HL (head length), TrL (trunk length), TaL (tail length), SL (standard length).

Voucher	AMS I. 49510-001	AMS I.47267-001	AMS I.47267-002
Type	holotype	paratype	paratype
Gender	male	female	female
Trunk rings	18	18	18
Tail rings	68	70	71
Subdorsal rings	19	20	20
Dorsal-fin origin	7^th^ trunk ring	6^th^ trunk ring	7^th^ trunk ring
Dorsal-fin rays	45	43	43
Pectoral-fin rays	18	13	13
SL (mm)	145.5	136.3	138.2
SnD/SnL	7.6	7.4	7.2
SnL/HL	63.2	65.5	64.5
SnL/TrL	36.1	39.3	35.8
HL/TrL	57.1	60.0	55.6
HL/SL	14.0	15.1	15.5
Trl/SL	24.5	25.2	27.9
Tal/SL	61.9	60.4	56.7

#### Colouration in life.

In life, *S. harastii* exhibits red background colouration with unique colour patterns: dorsum of snout with large, irregular pale white spots; sides of head and anterior trunk rings with large, irregular pale white or red spots or with diffuse pale white markings; sexual dimorphic markings with venter of first trunk ring exhibiting distinct red elongated spots in longitudinal row on midline, almost forming a stripe, present in males (Figs 1, 3, 5); and venter of anterior trunk rings lighter than sides and dorsum with a large cluster of distinct red spots extending posteriad from second trunk ring in males (Figs 1, 3, 5) whereas few scattered small red spots are present in the females (AMS I.47267). In alcohol, head and body background colour typically uniformly pale red (Fig. 1). Fins hyaline.

#### Etymology.

This species is named after David Harasti, one of the first to recognize *S. harastii* as being a new species, for recognition of his efforts towards conservation of Syngnathidae in Australia, and for being an aficionado extraordinaire of his beloved genus *Stigmatopora*. David has stated he counts green pipefish to fall asleep. Harasti’s Pipefish and the Red Wide-bodied Pipefish are proposed here as the common names for *S. harastii*.

#### Distribution and habitat.

*Stigmatopora harastii* is currently known to occur in central NSW, Australia from only three localities, including Botany Bay, Shellharbour, and Jervis Bay (Fig. 5). The paratypes and holotype described herein were collected separately at the scuba dive site referred to as The Steps between 2017 and 2020, respectively, at a depth of 11–12 meters at the southern entrance to Botany Bay, which is located within the Kamay Botany Bay National Park at Kurnell. The topography above and below the water at the type locality and the other localities is consistent for the Sydney Basin bioregion in the central eastern coast of Australia, which covers an area between Newcastle in the north to Bateman’s Bay in the south (Andrew 1999), and is comprised of weathered sandstone cliffs and flat intertidal platforms (Fig. 6). Subtidally, the rocky reefs typically have large undercut benches that are interspersed with large boulders and gutters. The underwater habitat closest to shore is composed of large boulders that are mostly devoid of sessile growth (Fig. 6), which is followed by sandy bottom at a depth of approximately 10–15 metres. At this depth, the sandy bottom is littered with large boulders, which are covered in prolific sessile growth, including ascidians, bryozoans, and sponges, and interspersed with beds of kelp (*Ecklonia*). Fucoid algae, and seagrass habitat comprising species that occur commonly in NSW coastal and estuarine areas such as *Posidonia australis* and *Zostera capricorni*, were recorded as absent. Along the sand edge of the entrance, small isolated clumps of red algae appearing to be of the family *Gracilariaceae* Nägeli, 1847, and possibly of the species *Crassiphycus secundatus* (Harvey) Gurgel, J.N. Norris & Fredericq, 2018, were attached to flat rocky substrate that was usually covered in sand. The red algae mostly appeared to occur at low densities as single or one to three adjoining clumps and widely dispersed throughout the habitat. Fluctuations in density of red algae was observed over many dives in the same area by the second author.

**Figure 7.**
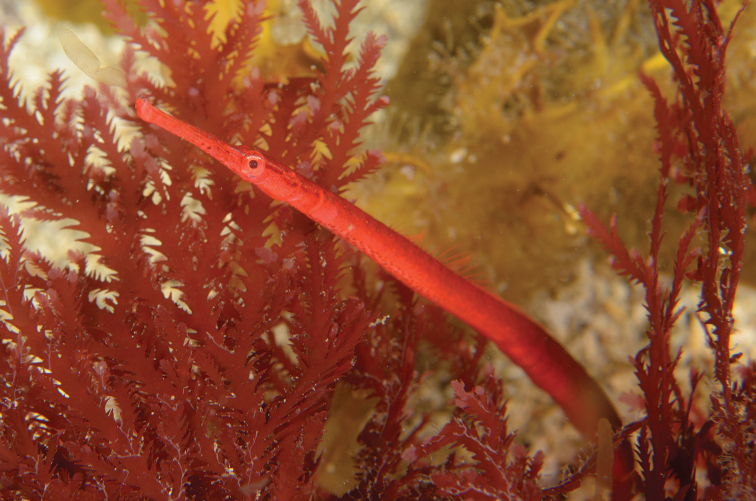
*Stigmatopora harastii* in situ, male, The Leap, Kurnell, Botany Bay, NSW, Australia, 12 meters depth, 03 October 2018 (photograph: Andrew Trevor-Jones).

Single individuals or male-female pairs of *S. harastii* were observed to be closely associated with the red algae; however, they were infrequently detected and present in only one isolated clump of red algae among all the other clumps in the close vicinity. Multiple dives by the second author between the collection of the type specimens at The Steps in the area where the type specimens were collected, as well as at other nearby large areas with red algae did not consistently detect the presence of *S. harastii*. Most times individuals were absent or only a single individual was found, possibly suggesting fluctuations in the abundance of the red algae with which *S. harastii* associates. Individuals were oriented vertically or at an angle and extremely well-camouflaged within the red algae, the distal third of their tails clasped around single fronds. The habitat was subject to strong surge in which individuals of *S. harastii* and the red algae together were observed swaying in unison with the surge (https://vimeo.com/229093467). The second author has observed on occasion active individuals swimming from one clump of red algae to another or feeding actively just outside the red algae. Feeding behaviour appears to be similar to other *Stigmatopora* species, with individuals darting out from the cover of their alga to capture food such as small copepods and shrimp. Individuals have been also observed nearby between the Steps and the dive site locally referred to as the Leap, one of which was associated with another species of red algae appearing to be of the genus *Gracilaria* Greville, 1830 (Fig. 7).

**Figure 8.**
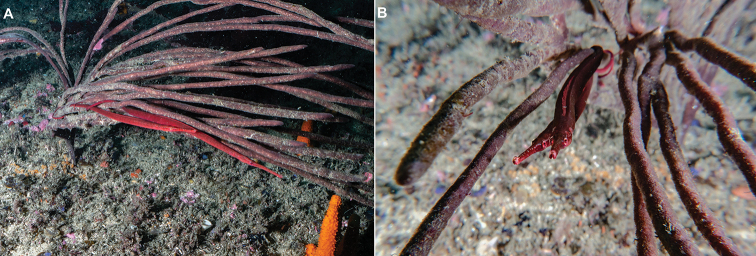
*Stigmatopora harastii* in situ, male-female pair **A** lateral view **B** anterior view, Minmi Trench, Botany Bay, NSW, Australia, 18 meters depth, 17 February 2019 (photographs: Duncan Heuer).

**Figure 9.**
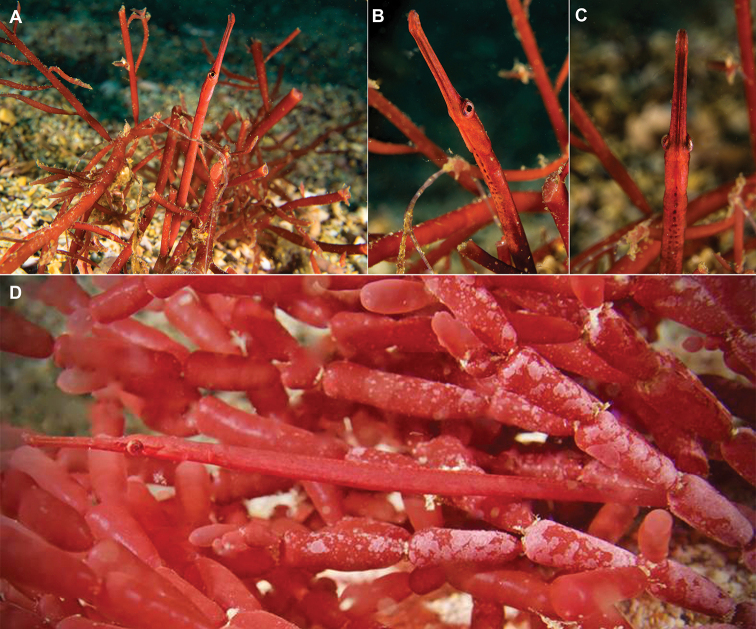
*Stigmatopora harastii* in situ **A–C** male **D** female, The Gutter, Bass Point, Shellharbour, NSW, Australia, 18 meters depth, 17 Feb 2017 (photographs: Craig Taylor).

*Stigmatopora harastii* was also observed at the dive site locally referred to as the Minmi Trench, located at the northern headland of Botany Bay, that consists of a flattish reef with small to large boulders at about 16 meters depth, which then drops off to 22–23 meters depth. A male-female pair was observed associating with a finger sponge appearing to be a member of the family *Callyspongiidae* Laubenfels, 1936 at 18 meters depth (Fig. 8). Photographs of *S. harastii* were also taken on January 2017 at the locality referred to as The Gutter at Bass Point, Shellharbour, NSW at 18 meters depth (Fig. 9). A male individual exhibiting the characteristic dimorphic colour pattern on its ventral trunk was observed in red algae appearing to be of the same species observed at Kurnell (Fig. 9A–C). Additionally, a female individual (Fig. 9D) was observed associating with a clump of bubble red algae appearing to be of a different species of red algae of the genus *Gracilaria*.

#### Morphological comparisons.

**Figure 10.**
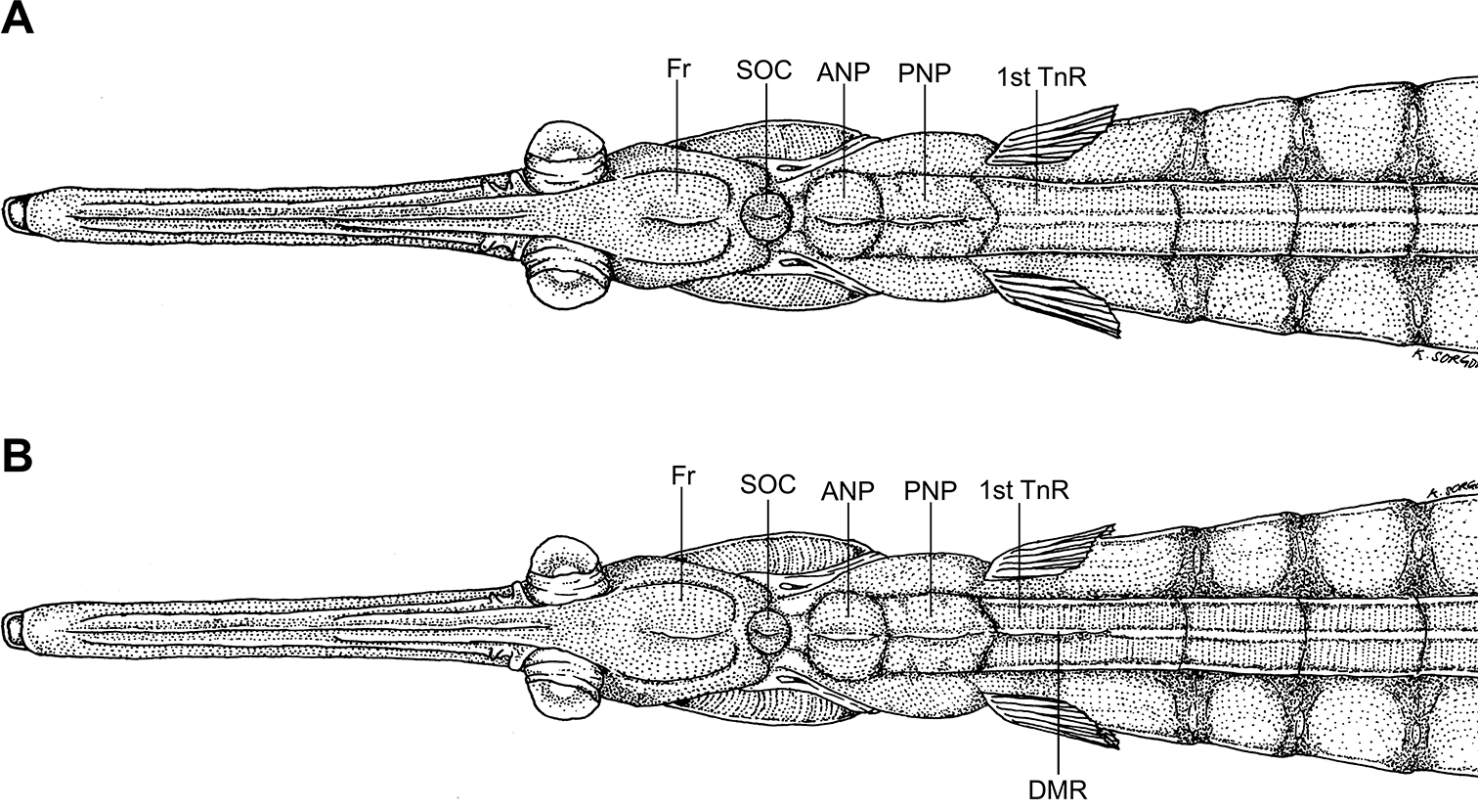
Comparison of the dorsal median ridge present on the head and first trunk ring in: **A***S. harastii* and **B***S. nigra*, AMS I.42611-009. Note the dorsal median ridge (DMR) extending into the first trunk ring in *S. nigra* versus ending on the posterior nuchal plate in *S. harastii*. Abbreviations: ANP, anterior nuchal plate; DMR, dorsal median ridge; Fr, frontal; PNP, posterior nuchal plate; SOC, supraoccipital; first TnR, first trunk ring (illustration by Kent Sorgon).

*Stigmatopora harastii* shares morphological synapomorphies with *S. argus*, *S. macropterygia*, *S. narinosa*, and *S. nigra*, including principle body ridges, dorsal-fin placement, distally attenuated, slender tail, and absence of caudal fin (Dawson 1982, 1985; Browne and Smith 2007). *Stigmatopora harastii* appears to be most similar to *S. nigra* (Dawson 1982: fig. 7) in meristics, snout length and shape, dorsal-fin origin on 6^th^–7^th^ trunk ring, presence of a distinct median longitudinal ridge between the opercle and pectoral-fin base, and lateral trunk ridge terminating on the first tail ring (Table 2). Even though all members of *Stigmatopora* share similar meristic and morphometric characters (Table 2; Dawson 1982, 1985; Browne and Smith 2007), they can be morphologically distinguished on the basis of a distinct but low dorsal median ridge present on the dorsum of the head and first trunk ring (Dawson 1982, 1985; Browne and Smith 2007). In *S. harastii*, the dorsal median ridge extends from the posterior third of the frontal to the supraoccipital, anterior, and posterior nuchal plates (Fig. 10) (versus the posterior third of the frontal to the supraoccipital, anterior, and posterior nuchal plates, and first trunk ring in *S. nigra* [Fig. 10; Dawson 1982: fig. 7]; the anterior and posterior nuchal plates in *S. argus* [Dawson 1982: fig. 2]; absence of low dorsal median ridge on head an first trunk ring in *S. macropterygia*; restricted to the supraoccipital in *S. narinosa*). *Stigmatopora harastii* also differs from *S. argus* and *S. macropterygia* in the termination of the lateral trunk ridge on the tail ring (first tail ring vs. 8^th^–20^th^ tail ring in *S. argus*; 22^nd^–35^th^ tail ring in *S. macropterygia*) and the presence of a median longitudinal ridge between the opercle and pectoral-fin base (vs. absence of longitudinal ridge). *Stigmatopora harastii* is distinguished from *S. narinosa* in the shape of the snout (long vs. medium length, laterally flattened, and dorsally elevated) and the presence of the longitudinal ridge between the opercle and pectoral-fin base (vs. absence of longitudinal ridge).

**Table 2.** Comparison of morphological characters between ​*S. harastii* and other members of *Stigmatopora*.

	*S. harastii*	*S. nigra*	*S. argus*	*S. macropterygia*	*S. narinosa*
Data source	This study	Dawson, 1982	Dawson, 1982	Dawson, 1982	Browne & Smith, 2007
Trunk rings	18	16–19	16–23	21–22	18
Tail rings	68–71	67–79	78–91	85–92	68
Dorsal-fin rays	43–45	35–47	37–64	63–74	37–45
Pectoral-fin rays	13–18	11–16	13–18	15–19	12–13
Dorsal-fin origin	6^th^–7^th^	5^th^–7^th^	9^th^–13^th^	8^th^–10^th^	5^th^–7^th^
Lateral trunk ridge reaches	1^st^ tail ring	Anal or 1^st^ tail ring	8^th^–20^th^ tail ring	22^nd^–35^th^ tail ring	2^nd^ tail ring
Snout length	long	long	long	long	medium, laterally flattened, dorsally elevated
Median dorsal ridge on head and first trunk ring	from frontal to posterior nuchal plate	from frontal to 1^st^ trunk ring	from anterior to posterior nuchal plate	absent	supraoccipital
Longitudinal ridge between opercle and pectoral-fin base	present	present	absent	absent	absent

#### Comparative colouration.

**Figure 11.**
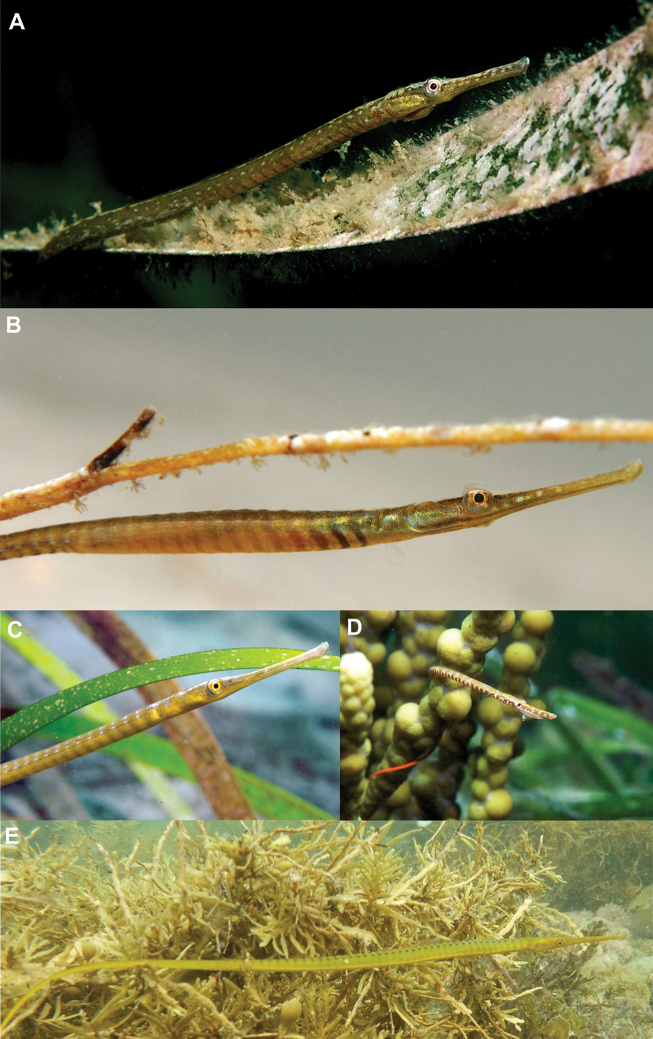
Fucoid algae and seagrass associating members of *Stigmatoporain situ***A***S. nigra*, male, Nelson Bay, NSW (photograph: David Harasti) **B***S. nigra*, female, Port Hughes, Gulf St Vincent, South Australia (photograph: David Muirhead) **C***S. argu*s, Port Hughes, Gulf St Vincent, South Australia (photograph: Graham Short) **D***S. narinosa*, Port Hughes, Gulf St Vincent, South Australia (photograph: Graham Short) **E***S. macropterygia*, Winstones Cove, North Island, New Zealand (photograph: Nick Shears).

The new species is most easily distinguished from *S. nigra* by features of the colour pattern in life (Figs 1–4, 6–9), including red background colour on the body (vs. base colour variably light brown to dark green in *S. nigra* [Fig. 11A, B]), and sexual dimorphic markings comprising (1) distinct red elongated spots in longitudinal row on midline, almost forming a stripe on venter of first trunk ring in the male and a smattering of red dots in the female (Figs 1–3, 6, 8B, C) (vs. small dots or striations crossing posterior area of snout, suborbital, and lower part of opercle on venter of head in *S. nigra* in the male and female [Fig. 11A, B; Dawson 1982: fig. 8]), and (2) a large cluster of distinct red spots extending posteriad from the second trunk ring on venter of the anterior trunk rings in the male and a smattering of red dots in the female (Figs 1–3, 7, 9B, C) (vs. presence of stripes between the trunk rings on the venter of all the trunk rings in *S. nigra*; pale stripes in the male, and dark stripes in the female, with the 2^nd^ and 3^rd^ trunk ring stripes darkest [Fig. 11A, B; Dawson 1982: fig. 8]). *Stigmatopora narinosa* exhibits two pairs of large black spots arranged in two rows, respectively, on venter of first trunk ring whereas dark transverse bands are present on the venter of each trunk and tail ring with anterior and dorsal margins of each ring white, forming thin white lines between each ring, resulting in appearance of a series of inverted saddles (Browne and Smith 2007). In contrast, *S. argus* and *S. macropterygia* exhibit no distinctive markings on venter of the head whereas venter of the trunk and tail in *S. argus* presents narrow dark bars between the rings on the whole trunk and anterior third of tail (vs. absence of markings on venter of trunk and tail in *S. macropterygia*; Fig. 11E; Dawson 1982).

*Stigmatopora harastii*, *S. nigra*, and *S. narinosa* share the presence of large, irregular pale white spots on dorsum of snout (vs. absence of white spots in *S. argus* and *S. macropterygia*). Finally, *S. harastii* and *S. nigra* share the presence of large, irregular pale white spots with scattering of small red dots, or diffuse pale white stripes, on sides of head and anterior superior trunk rings (vs. absence of colour markings on sides of head and anterior superior trunk rings in *S. argus* and *S. macropterygia*). In contrast, *Stigmatopora narinosa* exhibits a camouflage pattern of brown and white diffuse colouration on sides of head.

### Key to the species of *Stigmatopora*

**Table:** 

1	Dorsal-fin origin on 5–7^th^ trunk ring, lateral trunk ridge ends on anal, first, or second trunk ring	**2**
–	Dorsal-fin origin on 8^th^–13^th^ trunk ring	**5**
2	Snout long and slender	**3**
–	Snout shorter, laterally flattened, and dorsally elevated	**4**
3	Red dots on venter of anterior trunk rings (male and female)	***Stigmatopora harastii***
–	Black stripes on venter of anterior trunk rings (female)	***Stigmatopora nigra***
4	2 large transverse pairs of black dots on venter of trunk (female)	***Stigmatopora narinosa***
5	Lateral trunk ridge reaches 8^th^–23^rd^ tail ring, black spots on dorsum and venter of trunk	***Stigmatopora argus***
–	Lateral trunk ridge reaches 22^nd^–35^th^ tail ring, no distinctive markings on venter of trunk	***Stigmatopora macropterygia***

### Habitat preferences

*Stigmatopora harastii* inhabits semi-exposed bay entrances and ocean embayments in which the underwater terrain is characterised by sandy areas interspersed with boulders and hard flat reefs. Fucoid algae, and seagrass species that occur commonly in NSW coastal and estuarine areas such as *Posidonia australis* and *Zostera capricorni*, were absent but were recorded within the shallow areas of the bays or in nearby adjacent bays (Larkum and West 1990; Bell et al. 1992; Creese et al. 2009; Griffiths 2010; Parkinson and Booth 2016). Individuals and pairs of *S. harastii* were observed in close association with a species of finger sponge appearing to a member of the family *Callyspongiidae* Laubenfels, 1936 (Fig. 7) and several different species of red algae appearing to be of the family Gracilariaceae in the genera *Crassiphycus* and *Gracilaria* (Figs 4, 5, 7, 8). *Stigmatopora harastii* was not observed associating with any of the other numerous species of small to large sponges or tunicates present on the sessile-rich boulders nor within the canopy kelp *Ecklonia radiata*.

The unique habitat associations of *S. harastii* with a finger sponge and red algae differ markedly from the fucoid algae and seagrass associating members of *Stigmatopora*. *Stigmatopora argus*, *S. narinosa*, *S. nigra*, and *S. macropterygia* inhabit sheltered seagrass and algal beds in bays and estuaries throughout southern Australia and New Zealand, respectively. In NSW, the congenerics *S. nigra* and *S. argus* occur in sympatry in seagrass beds and form an abundant component of the inshore ichthyofauna (Steffe et al. 1989; Parkinson and Booth 2016). However, these species were not observed at the three localities in which *S. harastii* was found, which is most likely due to the absence of preferred seagrass and fucoid algae habitat. Similarly, in South Australia, the congenerics *S. argus*, *S. narinosa*, and *S. nigra* occur in sympatry and form an abundant component of the inshore ichthyofauna in which *S. argus* generally prefers to associate with the large seagrass genus *Posidonia*, *S. nigra* with the smaller seagrass genus *Zostera*, and *S. narinosa* with the fucoid corkweed *Scaberia agardhii*, *Posidonia*, and *Zostera* (Dawson 1982; Browne and Smith 2007). During a survey of the ichthyofauna at Smith Bay, Kangaroo Island (https://www.ausocean.org/cp/smithbay), located south of the Fleurieu and Yorke Peninsulas, the first author has observed *S. nigra* in small beds of *Zostera* to depths of 25–30 meters. It was not observed associating with the small species of sponges nor small clumps of red algae of unknown identification that were recorded in the habitat, which is characterised by flat sandy areas with small beds of *Zostera*, clumps of red algae, sponges, and scallops. Lastly, the New Zealand endemic *S. macropterygia* has been recorded in canopy beds of *Ecklonia*, *Zostera* down to 10 meters depth, and fucoid algae of the genera *Carpophyllum* and *Cystophora* (Dawson 1982; G. Short 2017, pers. obs.).

### COI Genetic Distances

Table 3 shows the genetic distance analysis at the COI gene (uncorrected p distances) between *S. harastii*, and the previously sequenced specimens of *S. argus* and *S. narinosa* (Hamilton et al. 2017) and the newly sequenced specimen of *S. macropterygia* from this study. *Stigmatopora harastii* differs from *S. argus* by 9.8%, from *S. nigra* by 14.6%, *S. narinosa* by 10.7%, and *S. macropterygia* by 10.1%. Reported mtDNA clock rates of approximately 1.2% per million years in marine teleosts (Reece et al. 2010) indicate divergence between *S. harastii* and *S. nigra* approximately 12.2 million years ago.

**Table 3.** Uncorrected genetic distances (*p*-distances) summary between *S. harastii* and other members of *Stigmatopora* based on cytochrome c oxidase I (COI) sequences analysed in this study.

	GenBank	Species	1	2	3	4	5
1	MK542828	*Stigmatopora harastii*					
2	KY066148	*Stigmatopora argus*	0.098				
3	MK552117	*Stigmatopora macropterygia*	0.101	0.054			
4	KY066149	*Stigmatopora narinosa*	0.107	0.115	0.115		
5	KY066150	*Stigmatopora nigra*	0.146	0.126	0.156	0.148	

## Discussion

Here we consider *S. harastii* as a valid species due to its morphological characters of the head, distinct sexual dimorphic markings on the ventral trunk rings, and genetic divergence from its congeners. We have identified a subtle but useful diagnostic morphological character consisting of a median dorsal ridge spanning the frontal, supraoccipital, and anterior and posterior nuchal plates that differentiate *S. harastii* from the superficially similar *S. nigra* (Figs 1, 9). In the previous diagnoses of *S. argus*, *S. macropterygia*, and *S. nigra* (Dawson 1982), the median dorsal ridge present on the neurocranial bones was cited, however its importance as a diagnostic character to differentiate between members of the genus was not recognised. The diagnosis of *S. narinosa* did not include this morphological character in the description (Browne and Smith 2007). Unequivocally, the most noticeable external features of *S. harastii* are the distinct sexual dimorphic red spotted markings on the ventral trunk rings in the male (Figs 1, 3, 6, 8), which differ markedly from the distinct markings exhibited on the ventral trunk rings by its congeners (Dawson 1982; Browne and Smith 2007). Typically, general body colouration in syngnathids is not considered a reliable feature for diagnosis (Lourie et al. 2016; Short et al. 2018), however, the presence of distinctive colour patterns on the venter of the anterior trunk rings is shared with the genus *Corythoichthys*, a sister species to *Stigmatopora* (Hamilton et al. 2017), in which all members bear diagnostic dark markings on the venter of anterior trunk rings that aid in their species identification.

The unique habitat associations of *S. harastii* with a finger sponge and red algae differ markedly from the fucoid algae and seagrass associating members of *Stigmatopora*. Even though no close association has been reported between fish and red algae in the literature until this study, Tyler and Böhlke (1972) documented 39 species of fish in the Caribbean known to have some association with sponges, and categorised sponge-dwelling fish as either (1) morphologically specialised obligate sponge dwellers, (2) morphologically unspecialised obligate sponge dwellers, (3) facultative sponge dwellers, or (4) fortuitous sponge dwellers. Facultative sponge dwellers in category 3 spend part of their lives on or in sponges, but have been observed in other types of habitat. Finally, fortuitous sponge dwellers in category 4 comprise a variety of families, all of which are known to occupy a wide variety of habitat types. The adult individuals of *S. harastii* that were observed during this study probably can be classified in category 3 as facultative finger sponge and red algae-dwellers, since *S. harastii* encountered at the type locality and other known localities only associates with one species of finger sponge (Fig. 7) and with at least three species of red algae (Figs 3, 4, 6, 8). It may prefer these habitat types since they provide many finger-like protuberances that allow for tail grasping and orienting their body in parallel to the finger sponge branches or red algae fronds, respectively, behaviours similarly seen in other members of *Stigmatopora* in fucoid algae and seagrass. *Stigmatopora harastii* has not been observed in the brown kelp *Ecklonia radiata* that is abundantly present near the red algae and finger sponge in their known localities, which may be due to the negative effects of the kelp canopy on feeding behaviour with respect to facilitating access in and out of the kelp habitat into the water column where they feed.

Other species of syngnathids form a component of the inshore ichthyofauna at the type locality where the authors and underwater photographers have recorded the following (1) seahorse species: *Hippocampus abdominalis*, *H. histrix*, *H. kelloggi*, and *H. whitei*; (2) pipefish species: *Festucalex cinctus*, *Heraldia nocturna*, *Lissocampus runa*, *Maroubra perserrata*, *Notiocampus ruber*, and *Trachyramphus bioarctatus*; (3) pygmy pipehorse species: *Idiotropiscis lumnitzeri*; and (4) weedy seadragon species: *Phyllopteryx taeniolatus*. *Hippocampus abdominalis*, *H. kelloggi*, and *H. whitei* have been observed associating with various species of sponges, as well as kelp and large tunicates (*Pyura spinifera*), and in a wide variety of habitat types at other localities in NSW (Harasti et al. 2010, 2012; Harasti 2014, 2015, 2017), including soft coral, seagrass, and shark nets, therefore placing them in category 4 as fortuitous habitat dwellers. Similarly, pairs of *Idiotropiscis lumnitzeri* has been observed on the large boulders using various species of sponges and soft corals as holdfasts, as well as bryozoans and calcified algae.

Habitat structural complexity plays an important role in shaping populations, community dynamics, and distribution of seagrass associating pipefish (Steffe et al. 1989; Connolly 1994; Jenkins et al. 1997; Jenkins and Wheatley 1998; Kendrick and Hyndes 2003; Curtis and Vincent 2005; Sanchez-Camara et al. 2006; Masonjones et al. 2010; Rose and Dixson 2010; Müller and Erzini 2017). *Stigmatopora harastii* likely has a wider distribution within NSW, southern Australia, and possibly New Zealand, where it remains undetected due to its preferred depth range, remarkable crypsis within its preferred habitat, and apparent low density of finger sponge and red algae occurring on the large boulders and flat rocky substrate, respectively. Its occurrence further north and south of central NSW may be confirmed by further sampling and by a better understanding of the distribution of the finger sponge and red algae with which it associates.

## Supplementary Material

XML Treatment for Stigmatopora harastii

## References

[B1] Andrew N (1999) Under Southern Seas: the ecology of Australia’s rocky reefs. UNSW Press, Sydney, 238 pp.

[B2] Bell JD Ferrell DJ McNeill SE Worthington DG (1992) Variation in assemblages of fish associated with deep and shallow margins of the seagrass *Posidonia australis*. Marine Biology 114(4): 667–676. 10.1007/BF00357264

[B3] Browne RK Smith K (2007) A new pipefish *Stigmatopora narinosa* (Syngnathidae) from South Australia. Memoirs of Museum Victoria 64: 1–6. 10.24199/j.mmv.2007.64.1

[B4] Browne RK Baker JL Connolly RM (2008) Syngnathids: seadragons, seahorses and pipefish. In: Shepherd SA Kirkegaard I Harbison P Jennings JT (Eds) Natural History of Gulf St Vincent.Royal Society South Australia, Adelaide, 162–176.

[B5] Connolly RM (1994) A comparison of fish assemblages from seagrass and unvegetated areas of a southern Australian estuary. Marine and Freshwater Research 45(6): 1033–1044. 10.1071/MF9941033

[B6] Creese RG Glasby TM West G Gallen C (2009) Mapping the Habitats of NSW Estuaries. Industry & Investment NSW Fisheries Final Report Series 113. New South Wales, Australia, 95 pp.

[B7] Curtis JM Vincent AC (2005) Distribution of sympatric seahorse species along a gradient of habitat complexity in a seagrass-dominated community. Marine Ecology Progress Series 291: 81–91. 10.3354/meps291081

[B8] Dayton PK Robilliard GA Paine RT Dayton LB (1974) Biological accommodation in the benthic community at McMurdo Sound. Antarctica Ecological Monographs 44(1): 105–128. 10.2307/1942321

[B9] Dawson CE (1977) Review of the pipefish genus *Corythoichthys* with description of three new species. Copeia 1977: 295–338. 10.2307/1443912

[B10] Dawson CE (1982) Review of the Indo-Pacific pipefish genus *Stigmatopora* (Syngnathidae). Records of the Australian Museum 34(13): 575–605. 10.3853/j.0067-1975.34.1982.243

[B11] Dawson CE (1985) Indo-Pacific Pipefishes (Red Sea to the Americas). Allen Press Inc., Lawrence, 230 pp.

[B12] Ferrell DJ McNeill SE Worthington DG Bell JD (1993) Temporal and spatial variation in the abundance of fish associated with the seagrass *Posidonia australis* in south-eastern Australia. Marine and Freshwater Research 44(6): 881–899. 10.1071/MF9930881

[B13] Fricke R Eschmeyer WN van der Laan R (2020) Eschmeyer’s Catalog of Fishes: Genera Species References. http://researcharchive.calacademy.org/research/ichthyology/catalog/fishcatmain.asp [Electronic version accessed 30 Jul 2020]

[B14] Griffiths S (2010) Diversity and distribution of fishes in an intermittently open coastal lagoon at Shellharbour New South Wales. Wetlands Australia 18(1): 13–24. 10.31646/wa.226

[B15] Hamilton H Saarman N Short G Sellas AB Moore B Hoang T Grace CL Gomon M Crow K Simison WB (2017) Molecular phylogeny and patterns of diversification in Syngnathid fishes. Molecular Phylogenetics and Evolution 107: 388–403. 10.1016/j.ympev.2016.10.003 27989632

[B16] Harasti D Glasby TM Martin‐Smith KM (2010) Striking a balance between retaining populations of protected seahorses and maintaining swimming nets. Aquatic Conservation: Marine and Freshwater Ecosystems 20(2): 159–166. 10.1002/aqc.1066

[B17] Harasti D Martin‐Smith K Gladstone W (2012) Population dynamics and life history of a geographically restricted seahorse *Hippocampus whitei*. Journal of Fish Biology 81(4): 1297–1314. 10.1111/j.1095-8649.2012.03406.x 22957871

[B18] Harasti D (2014) The biology ecology and conservation of White’s seahorse *Hippocampus whitei*. Doctoral dissertation.

[B19] Harasti D (2015) Range extension and first occurrence of the thorny seahorse *Hippocampus histrix* in New South Wales Australia. Marine Biodiversity Records 8: e49. 10.1017/S1755267215000263

[B20] Harasti D (2017) Southwards range extension of the great seahorse (*Hippocampus kelloggi*. Jordan & Snyder 1901) in Australia. Journal of Applied Ichthyology 33(5): 1018–1020. 10.1111/jai.13414

[B21] Howard RK Koehn JD (1985) Population dynamics and feeding ecology of pipefish (Syngnathidae) associated with eelgrass beds of Western Port Victoria. Marine and Freshwater Research 36(3): 361–370. 10.1071/MF9850361

[B22] Kendrick AJ Hyndes GA (2003) Patterns in the abundance and size-distribution of syngnathid fishes among habitats in a seagrass-dominated marine environment. Estuarine Coastal and Shelf Science 57(4): 631–640. 10.1016/S0272-7714(02)00402-X

[B23] Jenkins GP May HMA Wheatley MJ Holloway MG (1997) Comparison of fish assemblages associated with seagrass and adjacent unvegetated habitats of Port Phillip Bay and Corner Inlet Victoria Australia with emphasis on commercial species. Estuarine Coastal and Shelf. Science 44(5): 569–588. 10.1006/ecss.1996.0131

[B24] Jenkins GP Wheatley MJ (1998) The influence of habitat structure on nearshore fish assemblages in a southern Australian embayment: comparison of shallow seagrass reef-algal and unvegetated sand habitats with emphasis on their importance to recruitment. Journal of Experimental Marine Biology and Ecology 221(2): 147–172. 10.1016/S0022-0981(97)00121-4

[B25] Larkum AWD West RJ (1990) Long-term changes of seagrass meadows in Botany Bay Australia. Aquatic Botany 37(1): 55–70. 10.1016/0304-3770(90)90064-R

[B26] Manning CG Foster SJ Harasti D Vincent AC (2018) A holistic investigation of the ecological correlates of abundance and body size for the endangered White’s seahorse *Hippocampus whitei*. Journal of Fish Biology 93(4): 649–663. 10.1111/jfb.13745 29971766

[B27] Masonjones HD Rose E McRae LB Dixson DL (2010) An examination of the population dynamics of syngnathid fishes within Tampa Bay Florida USA. Current Zoology 56(1): 118–133. 10.1093/czoolo/56.1.118

[B28] Müller C Erzini K (2017) Interspecific differences in habitat selection of syngnathids in the Ria Formosa lagoon Portugal. Estuarine Coastal and Shelf Science 189: 235–242. 10.1016/j.ecss.2017.03.022

[B29] Parkinson KL Booth DJ (2016) Rapid growth and short life spans characterize pipefish populations in vulnerable seagrass beds. Journal of Fish Biology 88(5): 1847–1855. 10.1111/jfb.12950 27005315

[B30] Pollard DA (1984) A review of ecological studies on seagrass-fish communities with particular reference to recent studies in Australia. Aquatic Botany 18: 3–42. 10.1016/0304-3770(84)90079-2

[B31] Pollard DA (1994) A comparison of fish assemblages and fisheries in intermittently open and permanently open coastal lagoons on the south coast of New South Wales south-eastern Australia. Estuaries 17: 631–646. 10.2307/1352411

[B32] Rose E Dixson DL (2010) An examination of the population dynamics of syngnathid fishes within Tampa Bay Florida. USA Current Zoology 56(1): 118–133. 10.1093/czoolo/56.1.118

[B33] Sanchez-Camara J Booth DJ Murdoch J Watts D Turon X (2006) Density habitat use and behaviour of the weedy seadragon *Phyllopteryx taeniolatus* (Teleostei: Syngnathidae) around Sydney New South Wales Australia. Marine and Freshwater Research 57(7): 737–745. 10.1071/MF05220

[B34] Steffe AS Westoby M Bell JD (1989) Habitat selection and diet in two species of pipefish from seagrass: sex differences. Marine Ecology Progress Series 55: 23–30. 10.3354/meps055023

[B35] Tyler JC Böhlke JE (1972) Records of sponge-dwelling fishes primarily of the Caribbean. Bulletin of Marine Science 22(3): 601–642.

[B36] WoRMS Editorial Board (2020) World Register of Marine Species. https://www.marinespecies.org [Accessed 30 July 2020]

